# Supplementary material for: The Evolution and Characterization of the RNA Interference Pathways in Lophotrochozoa
Source: Genome Biol Evol. 2024 May 7;16(5):evae098. doi: 10.1093/gbe/evae098 (PMC11114477; doi:10.1093/gbe/evae098)
Supplement: evae098_Supplementary_Data [file evae098_supplementary_data.zip › Supplementary_materials.zip!/selected_miRNAs/NoGe_selected_miRNAs/known_miRNA_families_pdf_files/NMRB01000380.1_Notospermus_geniculatus_strain_Ushimado_scaffold380,_whole_genome_shotgun_sequence_11915.pdf]

Provisional ID	:	NMRB01000380.1_Notospermus_geniculatus_strain_Ushimado_scaffold380,_whole_genome_shotgun_sequence_11915
Score total	:	2359.8
Score for star read(s)	:	3.9
Score for read counts	:	2350.9
Score for mfe	:	2
Score for randfold	:	0
Score for cons. seed	:	3
Total read count	:	4623
Mature read count	:	4485
Loop read count	:	0
Star read count	:	138

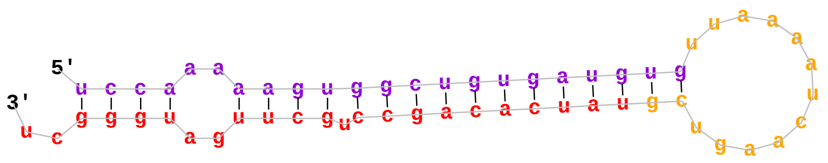

Genome coordinates: NMRB01000380.1 228038-228097

pre-miRNA sequence:
UCCAAAAAGUGGCUGUGAUGUGUCCAAAAAGUGGCUGUGAUGUGUUAAAAUCAAGUCGUAUCACAGCCUGCUUGAUGGGCU

Mature sequence: UAUCACAGCCUGCUUGAUGGGCU

Aligned mature sequence of the *N. geniculatus* pre-miRNA with the top 5 hits from the BLAST search against the pre-miRNA mirGeneDB set:

```
                        ***************
Nge_Mir-2-o18           uaucacagccugcuugaugggcu
Efe-Mir-2-o46_3p        uaucacagccugcuuggauuaug
Lan-Mir-2-o73_3p        uaucacagccugcuuugaugaga
```
